# Supplementary material for: Human Bone Marrow Mesenchymal Stem Cells Promote the M2 Phenotype in Macrophages Derived from STEMI Patients
Source: Int J Mol Sci. 2023 Nov 13;24(22):16257. doi: 10.3390/ijms242216257 (PMC10671615; doi:10.3390/ijms242216257)
Supplement: Supplementary file 1 [file ijms-24-16257-s001.zip › ijms-2687469-supplementary.pdf]

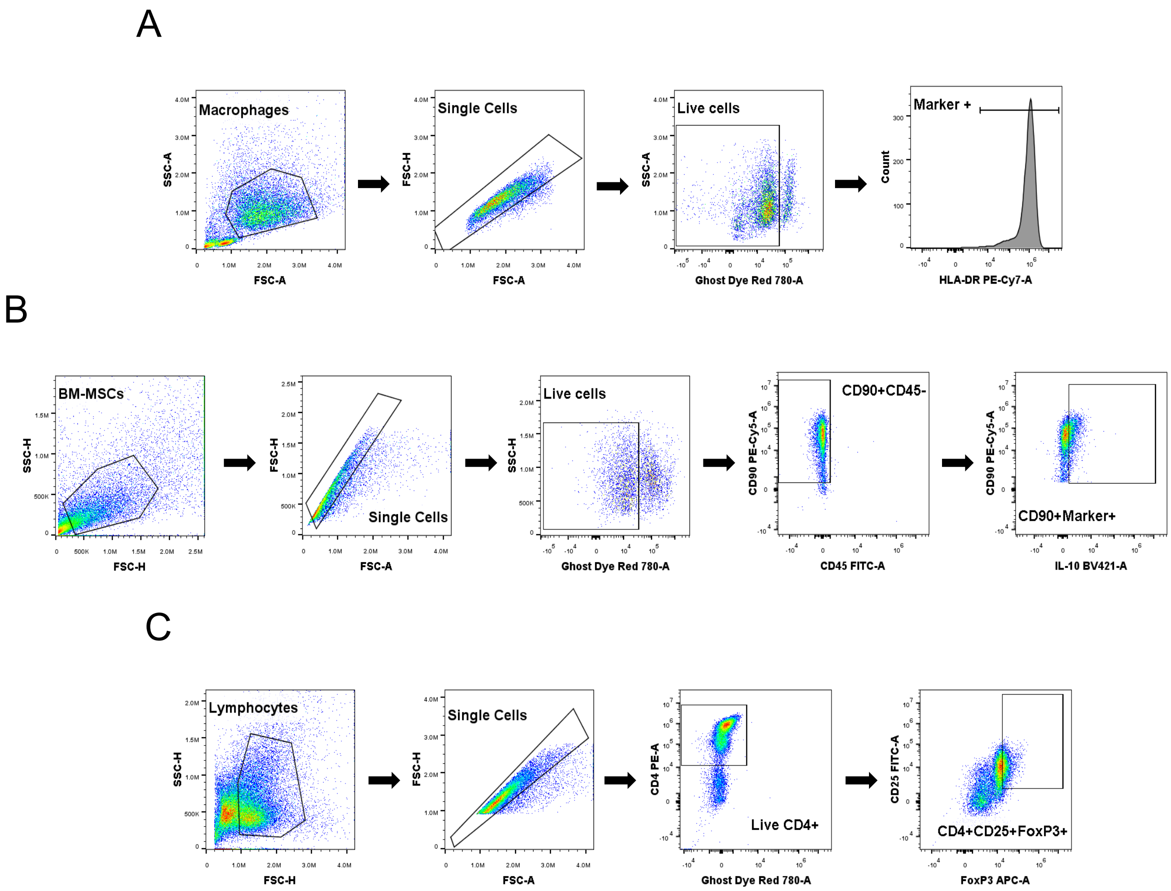

Figure S1. Flow cytometric strategy. A) Representative flow cytometry strategy. A) For macrophages, using SSC-A and FSC-A plots, the region of interest was identified from a plot of singlets (FSC-A vs. FSC-H) where double events were excluded and Ghost Dye Red 780-stained dead cells were excluded. From the live cell region, we analyzed different markers. This strategy was used for analyzing M1 and M2 macrophages. B) For BM-MSCs, using SSC-H and FSC-H, the region of interest was identified, and double events as well as dead cells were excluded. From the live cell region, BM-MSCs were identified as CD45-negative and CD90-positive cells (CD90+CD45-). CD90+CD45- BM-MSCs were analyzed for the expression of intracellular cytokines, such as IL-10. C) Gating strategy for determining regulatory T cells. First, using SSC-H and FSC-H, the region of interest was identified, and double events as well as dead cells were excluded. From the live cell region, a subset of CD4-positive cells (CD4+) was identified. Then, the regulatory T cells were identified as CD4+CD25+FoxP3+ cells.
